# Cytokine-enhanced cytolytic activity of exosomes from NK Cells

**DOI:** 10.1038/s41417-021-00352-2

**Published:** 2021-07-27

**Authors:** Yutaka Enomoto, Peng Li, Lisa M. Jenkins, Dimitrios Anastasakis, Gaelyn C. Lyons, Markus Hafner, Warren J. Leonard

**Affiliations:** 1 Laboratory of Molecular Immunology, Immunology Center, National Heart, Lung, and Blood Institute, National Institutes of Health, Bethesda, MD 20892-1674 USA; 2 Laboratory of Cell Biology, Center for Cancer Research, National Cancer Institute, Bethesda, MD 20892 USA; 3 RNA Molecular Biology Group, National Institute of Arthritis and Musculoskeletal and Skin Diseases, NIH, Bethesda, MD 20892 USA

**Keywords:** Cytokines, Innate immunity

## Abstract

Natural killer (NK) cells play key roles in immune surveillance against tumors and viral infection. NK cells distinguish abnormal cells from healthy cells by cell–cell interaction with cell surface proteins and then attack target cells via multiple mechanisms. In addition, extracellular vesicles (EVs) derived from NK cells (NK-EVs), including exosomes, possess cytotoxic capacity against tumor cells, but their characteristics and regulation by cytokines remain unknown. Here, we report that EVs derived from human NK-92 cells stimulated with IL-15 + IL-21 show enhanced cytotoxic capacity against tumor cells. Major cytolytic granules, granzyme B and granzyme H, are enriched by IL-15 + IL-21 stimulation in NK-EVs; however, knockout experiments reveal those cytolytic granules are independent of enhanced cytotoxic capacity. To find out the key molecules, mass spectrometry analyses were performed with different cytokine conditions, no cytokine, IL-15, IL-21, or IL-15 + IL-21. We then found that CD226 (DNAM-1) on NK-EVs is enriched by IL-15 + IL-21 stimulation and that blocking antibodies against CD226 reduced the cytolytic activity of NK-EVs. We also show NK-EVs are taken up by target cells via macropinocytosis. Collectively, our findings elucidate the novel properties of NK-EVs and the mechanism of their incorporation into target cells.

## Introduction

Natural killer (NK) cells play important roles in immune surveillance of tumors or viral infection [1, 2]. NK cells are known to secrete a large amount of interferon-γ (IFN-γ) to augment T helper type 1 (T_H_1) immune responses and also directly kill tumor cells and virally infected cells [1–3]. NK cells express many types of receptors on their cell surface that help to distinguish abnormal from healthy cells [1, 3]. After recognizing target cells, NK cells induce the death of these cells via a number of mechanisms, mediated by Fas ligand (FasL), TRAIL, secretion of tumor necrosis factor-α (TNF-α), IFN-γ, perforin, and granzymes [1–3]. In addition, NK-EVs, including exosomes, have been reported to show cytotoxicity against tumor cells [4–7].

EVs are nano-sized membrane vesicles released from most cell types that are known to contain various cellular components, including proteins, DNAs, mRNAs, and microRNAs (miRNAs) [8]. For cell-to-cell communication, receptor–ligand interactions and many types of molecules including cytokines, chemokines, growth factors, and hormones are involved [9]. Interestingly, EVs have been reported to be taken up by and to transfer their contents to spatially separated target cells, changing their phenotype [8, 10–12]. Thus, EVs can serve as novel communication tools.

Immune cell-secreted EVs serve to regulate both the innate and acquired immune responses [8, 13]. In addition, recent studies have shown that EVs can influence antitumor immune responses [13–16]. For example, EVs derived from activated CD8^+^　T cells prevent tumor progression by targeting mesenchymal cells in the tumor microenvironment [14]. EVs derived from DCs also activate and enhance the cytotoxic activity of NK cells via HLA-B-associated transcript 3 (BAT3), TNF superfamily members (TNF, TRAIL, and FasL), and interleukin-15Rα (IL-15Rα) on their surfaces [13, 17–19].

Previous studies reported that NK-EVs express various NK-cell receptors and cytotoxic molecules and can mediate cytotoxic activity against tumor cells [4–6, 20]. Moreover, NK-EVs are present in human blood, and it was proposed that they may be involved in NK-mediated immune surveillance of primary tumors [5] and shown that they have utility in cancer therapy in a mouse model [6, 7]. Therefore, NK-EVs may prove to be useful for immunotherapy, but the critical cellular components in NK-EVs remain elusive, although proteins or miRNAs in the NK-EVs are potentially important. The cytotoxic activity of NK cells is enhanced by cytokine stimulation, which induces the expression of cytotoxic molecules [21–23], but the effect of cytokines on NK-EVs and the pathway for their uptake by target cells remains unclear.

In this study, we have investigated the effect of treatment of NK-92 cells with IL-15, IL-21, and IL-15 + IL-21 on the cytotoxic activities of EVs produced by the cells. NK-92 is a human NK cell line derived from a patient with malignant non-Hodgkin’s lymphoma and retains cytolytic activity against cancer cells. NK-92 is FDA-approved for cancer immunotherapy and has been investigated in clinical trials [24]. Along with IL-2, IL-4, IL-7, and IL-9, IL-15 and IL-21 are members of a family of cytokines that bind receptors that contain the common cytokine-receptor γ chain, γ_c_, and both of these cytokines can augment the proliferation and cytotoxic activity of NK cells [25]. Moreover, the combination of IL-15 + IL-21 has a synergistic effect on the clonal expansion and cytotoxic activity of CD8^+^ T cells, resulting in potent antitumor effects by these cells [25, 26], and in vivo antitumor activity by IL-21 is mediated in part by NK cells [27]. In addition, IL-21 synergizes with IL-15 to augment IFN-γ production in NK and T cells [28]. Interestingly, treatment with IL-15 + IL-21 enhanced the cytotoxic activity of EVs produced by these cells. Based on small RNA-seq and mass spectrometry, the profiles of the cellular components in NK-EVs are altered by cytokine stimulation of NK-92 cells. Among the proteins whose expression is enriched by IL-15 + IL-21 stimulation, we then found that CD226 (DNAM-1) is related to cytolytic activity of NK-EVs. We also found that NK-EVs are taken up by target cells via macropinocytosis, an endocytosis pathway. Taken together, these findings reveal that cytokine stimulation on NK-92 cells changes the cytotoxic activity and the character of NK-EVs, which induces up-regulation of CD226. Moreover, we have elucidated the pathway of uptake of NK-EVs by target cells.

## Materials and methods

### Cell culture

NK-92 cells were cultured in MyeloCult H5100 medium (STEMCELL) containing penicillin–streptomycin (Gemini Bio-Products) and 100 IU/ml of recombinant human IL-2. K562 and Jurkat cells were cultured in RPMI-1640 medium (Gibco) containing 10% fetal bovine serum (FBS) and penicillin–streptomycin. A549 and HeLa cells were cultured in DMEM (Gibco) containing 10% FBS and penicillin–streptomycin. For stimulation, NK-92 cells were washed with phosphate-buffered saline (PBS) and rested overnight in MyeloCult H5100 without IL-2 and then cultured in MyeloCult H5100 with 50 ng/ml of IL-15, IL-21, IL-15 + IL-21, or without any cytokine for 3 days, washed with PBS, and cultured in Advanced RPMI-1640 (Gibco) containing penicillin–streptomycin and 2 mM l-glutamine (Gemini Bio-Products) continuously without cytokine or with 50 ng/ml of IL-15, IL-21, IL-15 + IL-21 for 2 days.

### Isolation of NK-EVs

NK-92 cells were cultured in Advanced RPMI-1640 for 2 days before isolation of NK-EVs. The medium was collected and centrifuged at 2000*g* for 10 min at 4 °C. The supernatant was filtered with a 0.22-μm filter unit (Millipore) to remove cellular debris and then ultracentrifuged in a Beckman SW32Ti rotor at 100,000 g for 2 h at 4 °C. The pellets were washed with PBS, ultracentrifuged again, and resuspended in PBS. The recovered NK-EV protein was measured using the Micro BCA Protein Assay Kit (Thermo Fisher Scientific). The size distribution of NK-EVs was quantified by nanoparticle tracking analysis (NTA) with NanoSight NS300 (Malvern).

### Western blotting

Cells or NK-EVs were lysed with NP40 Cell Lysis Buffer containing protease and phosphatase inhibitor cocktail (Thermo Fisher Scientific). Cell lysates were incubated with Loading Dye (LI-COR) for 30 min under non-reducing conditions at 37 °C to detect CD63 as previously described [29] or with Loading Dye and 2-mercaptoethenol for 5 min at 95 °C to detect other proteins. Proteins were loaded onto NuPAGE gels (Invitrogen), transferred to membranes, and the membranes were incubated with 5% skim milk in TBS-T for 1 h at room temperature. The membranes were then incubated overnight at 4 °C with primary antibodies, anti-CD81, CD63 (Cosmo Bio, clone 12C4, 8A12, respectively), cytochrome *c*, granzyme B (Biolegend, clone 7H8.2C12, O94E6, respectively), β-actin, granzyme H (Cell Signaling Technology, catalog 4967, 18268, respectively), and α-tubulin (Santa Cruz, catalog sc-23948). Secondary antibodies, IRDye 680RD-conjugated anti-mouse IgG, or anti-rabbit IgG (LI-COR) were used at a 1:5000 dilution. The membranes were exposed to ODYSSEY CLx (LI-COR).

### Cytotoxic assay

Target cells were labeled with 0.2 μM CFSE (eBioscience). CFSE-labeled K562 or Jurkat cells (2 × 10^4^) were cultured in 96-well plates with NK-EVs for 12 or 24 h. CFSE-labeled A549 or HeLa cells (1 × 10^4^) were seeded onto 96-well plates the day before adding NK-EVs and then cultured with NK-EVs for 24 h. For experiments with blocking antibodies to CD226, NK-EVs were incubated with isotype control (Biolegend, clone 401402) or anti-CD226 antibodies (Abcam, clone DX11) [30, 31] for 30 min at 37 °C and then incubated with K562 cells. The final concentration of isotype control or anti-CD226 antibodies was 10 μg/ml. For examining the cytotoxicity of NK-92 cells, CFSE-labeled K562 cells (5 × 10^3^) were co-cultured with 5 × 10^4^ NK-92 cells for 4 h. Dead cells were measured by CFSE^+^/PI^+^ cells using a FACS Canto II flow cytometer.

### Quantitative reverse transcriptase PCR (qRT-PCR)

Total RNAs were extracted with TRIZOL Reagents (Thermo Fisher Scientific) and reverse transcribed using QuantiNova Reverse Transcription Kit (Qiagen). Quantitative real-time RT-PCR were performed on a CFX96 Real-Time System (Bio-Rad) using Taqman Assays (Thermo Fisher Scientific) and THUNDERBIRD Probe qPCR Master Mix (TOYOBO). RPL7 was used as an internal control. Quantitative real-time RT-PCR was performed in triplicate for each sample.

### Mass spectrometry analysis

The pellets of NK-EVs were solubilized in 5% SDS, 50 mM triethylammonium bicarbonate buffer (TEAB), pH 7.55 and the proteins reduced and alkylated with iodoacetamide. The proteins were then loaded onto S trap columns (Protifi) for overnight trypsin digestion at 37 °C, following the manufacturer’s instructions. The resultant peptides were desalted on a C18 spin column (Pierce) and lyophilized. Dried peptides were solubilized in 2% acetonitrile, 0.5% formic acid, 97.5% water for analysis on an Orbitrap Fusion (Thermo) mass spectrometer. Proteome Discoverer 2.3 (Thermo) was used to search the data against human proteins from the UniProt database using SequestHT. The Percolator node was used to score and rank peptide matches using a 1% false discovery rate. The mass spectrometry proteomics data have been deposited to the ProteomeXchange Consortium via the PRIDE partner repository with the dataset identifier PXD018982 and 10.6019/PXD018982.

### Gene Ontology (GO) analysis

GO analysis was performed with proteins enriched in NK-EVs by cytokine stimulation. ShinyGO v0.61 (http://bioinformatics.sdstate.edu/go/) was used to analyze proteins with a 0.05 *P* value cutoff, and the 30 most significant terms were shown.

### Small RNA-seq

NK-92 cells were washed with PBS and rested overnight in MyeloCult H5100 without IL-2. NK-92 cells were then cultured in MyeloCult H5100 with 50 ng/ml of IL-15, IL-21, IL-15 + IL-21, or without any cytokine for 3 days, washed with PBS, and cultured in Advanced RPMI-1640 (Gibco) containing penicillin–streptomycin and 2 mM l-glutamine (Gemini Bio-Products) continuously with 50 ng/ml of IL-15, IL-21, IL-15 + IL-21, or without any cytokines for 2 days. Total RNA was isolated from EVs and NK-92 cells with Direct-zol RNA Kits (Zymo Research). A small RNA cDNA library was prepared and then sequenced by Illumina HiSeq3000 machine as described previously [32]. The frequency represents the occurrences of a microRNA out of the total sequenced reads in that small RNA-seq library. The small RNA-seq data have been deposited to the GEO repository with the GEO accession number GSE150342.

### Prediction of target genes of miRNAs

Target genes of each miRNAs were predicted by miRTarBase (http://mirtarbase.mbc.nctu.edu.tw/php/index.php). Potential target genes that were examined by at least two evidence were listed.

### NK-EVs uptake assay

NK-EVs were labeled with PKH67 by using PKH67 Green Fluorescent Cell Linker Kit for General Cell Membrane Labeling (Sigma). NK-EVs or the same volume of PBS were incubated with 2 μM PKH67 for 5 min at room temperature and then washed with PBS three times on an Amicon Ultra 0.5 ml Centrifugal Filters. Labeled NK-EVs were resuspended with PBS. K562 cells were incubated with PKH67-labeled NK-EVs for 3, 6, or 24 h at 37 or 4 °C. In order to identify the pathway by which NK-EVs uptake occurs, K562 cells and Jurkat cells were preincubated with 75 μM EIPA (Sigma) diluted with DMSO or the same concentration of DMSO as vehicle control 30 min before incubation with PKH67-labeled NK-EVs to block macropinocytosis. PKH67^+^ cells were measured using a FACS Canto II flow cytometer. Viable (PI^−^) cells were gated to determine PKH67 expression.

### Confocal microscopy analysis

NK-EVs were labeled with PKH67 as described above. K562 cells were incubated with 5 μg of PKH67-labeled NK-EVs for 24 h at 37 °C. Cells were washed with PBS and prepared using a Cytospin 4 cytocentrifuge (Thermo Fisher Scientific). Cells were then fixed with 4% paraformaldehyde, and their nuclei were stained with DAPI (Thermo Fisher Scientific). After washing with PBS, cells were mounted with Fluoromount-G (Thermo Fisher Scientific) and images were obtained by an LSM880 microscope (Zeiss) equipped with ×63/1.4 oil objective.

### Statistical analysis

Unless otherwise stated, data are shown as means + SEM and were compared using two-tailed Student’s *t*-test. A value of *P* < 0.05 was taken to indicate statistical significance. Statistical analyses were performed using GraphPad Prism (GraphPad Software, USA).

## Results

### EVs derived from NK-92 cells possess cytotoxic activity

**Fig. 1:**
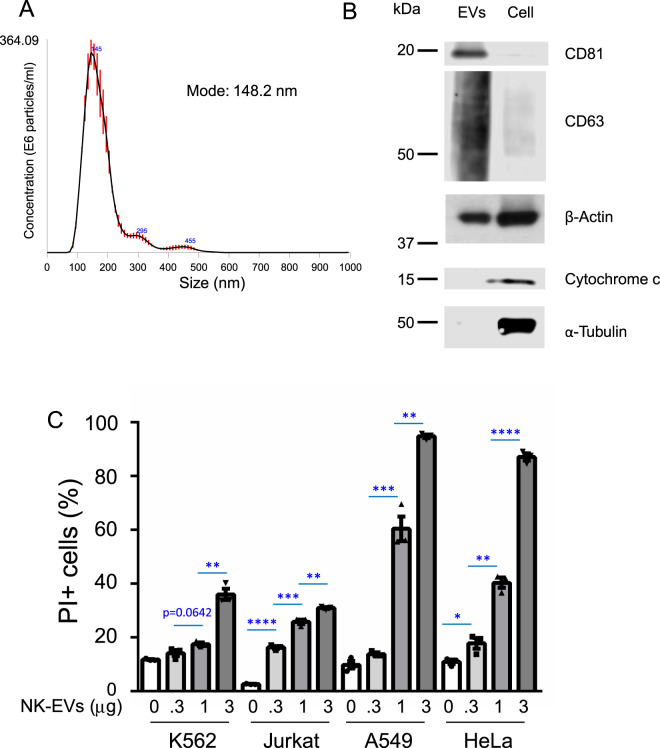
EVs derived from NK-92 cells possess cytotoxic activity. **A** The size distribution of NK-EVs was analyzed by nanoparticle tracking analysis (NTA). The data shown are merged data from three individual tracings. **B** Lysates of NK-EVs and cells were immunoblotted with antibodies to CD81, CD63, β-actin, cytochrome *c*, or α-tubulin. **C** CFSE-labeled K562 cells or Jurkat cells (2 × 10^4^) were cultured with 0, 0.3, 1, or 3 μg of NK-EVs for 24 h. CFSE-labeled A549 cells or HeLa cells (1 × 10^4^ cells) were seeded overnight and then cultured with 0, 0.3, 1, or 3 μg of NK-EVs for 24 h. The graphs represent the means + SEM of percentages of CFSE^+^/ PI^+^ cells. Data are shown as means + SEM of three samples. All data shown are representative of three independent experiments. *P < 0.05; **P < 0.01; ***P < 0.001; ****P < 0.0001.

We harvested the supernatant of NK-92 cells and isolated EVs by ultracentrifugation (see “Methods”). Using NTA, we confirmed that we successfully isolated NK-EVs of a size typical for exosomes [4, 6]: the average size (mode) of isolated NK-EVs from three tracings was 148.2 nm (Fig. 1A). Moreover, examination of isolated NK-EVs and whole-cell lysates from NK-92 cells by western blotting confirmed that CD81 and CD63, well known exosome markers, were enriched in the NK-EVs (Fig. 1B). Although β-actin was expressed by both NK-EVs and whole-cell lysates (Fig. 1B), cytochrome *c* and α-tubulin, which are highly enriched in cytoplasm, were at most minimally expressed by the NK-EVs (Fig. 1B). Thus, our cells and their derivative NK-EVs were distinctive in their composition, consistent with the characteristics of EVs reported previously [4, 33, 34].

To assess the biological properties of isolated NK-EVs, we measured cytolytic activity against K562, Jurkat, A549, and HeLa cell lines, which are derived from patients with chronic myelogenous leukemia, acute T cell leukemia, lung carcinoma, and adenocarcinoma of the cervix, respectively. We found that the NK-EVs possessed cytotoxic activity against each of these cell lines in a dose-dependent manner, with greater killing of the A549 and HeLa cells than of K562 and Jurkat cells (Fig. 1C) (Supplementary Fig. 1 shows a representative result, with multiple experiments summarized in Fig. 1C).

### NK-EVs are incorporated into target cells via macropinocytosis

**Fig. 2:**
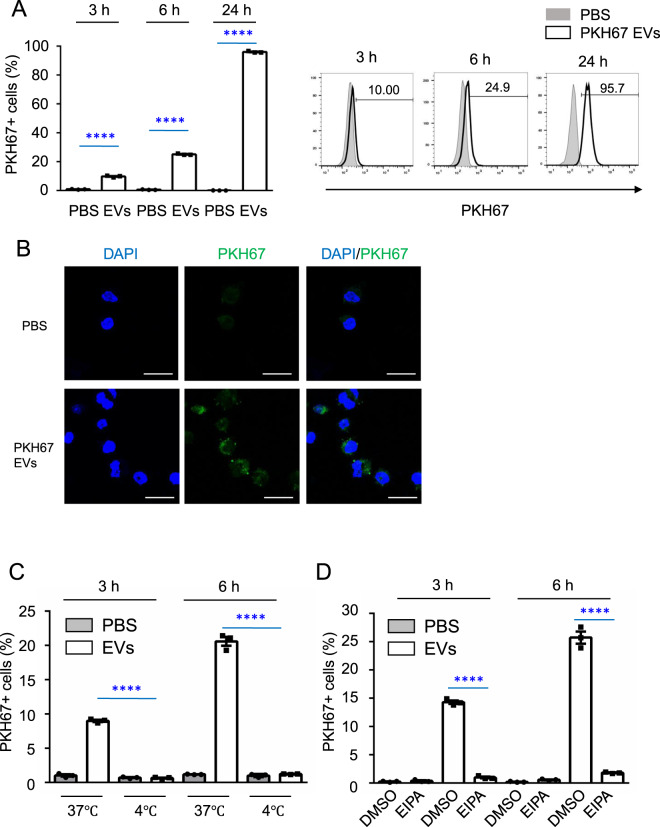
EVs derived from NK-92 cells are taken up by recipient cells via macropinocytosis. **A** K562 cells were incubated with PBS or 5 μg of PKH67-labeled NK-EVs for 3, 6, or 24 h at 37 °C. The percentage of PKH67^+^ K562 cells was determined by flow cytometry. The graphs represent the means + SEM of the percentage of PKH67^+^ K562 cells. Numbers indicate the percentage of PKH67^+^ cells. **B** K562 cells were incubated with PBS or 5 μg of PKH67-labeled NK-EVs for 24 h at 37 °C. Nuclei were stained with DAPI (scale bars, 20 μm). **C** K562 cells were incubated with PBS or 5 μg of PKH67-labeled NK-EVs for 3 or 6 h at 37 or 4 °C. The percentage of PKH67^+^ K562 cells were analyzed by flow cytometry. Viablility of cells was approximately 95% after incubation for 3 or 6 h at 4 °C. The graphs show the means + SEM of the percentage of PKH67^+^ K562 cells. **D** K562 cells were preincubated with DMSO or 75 μM EIPA for 30 min. Cells were then incubated with PBS or 5 μg of PKH67-labeled NK-EVs for 3 or 6 h. The percentage of PKH67^+^ K562 cells were analyzed by flow cytometry. The graphs represent the means + SEM of the percentage of PKH67^+^ K562 cells. Cell cultures were performed in triplicate. All data shown are representative of three independent experiments. ****P < 0.0001.

We next confirmed that NK-EVs were indeed taken up by recipient cells by incubating K562 cells with PKH67-labeled NK-EVs at 37 °C for 3, 6, or 24 h and then examining PKH67^+^ cells by flow cytometry. NK-EVs were gradually taken up by recipient cells, with most cells PKH67^+^ 24 h after incubation (Fig. 2A). We confirmed that PKH67-labeled NK-EVs were taken up by recipient K562 cells, as assessed at 24 h by confocal microscopy (Fig. 2B). To further investigate the pathway of NK-EVs-uptake by recipient cells, we incubated K562 cells with PKH67-labeled NK-EVs and found that NK-EVs were taken up by recipient cells at 37 °C but not at 4 °C (Fig. 2C). Since endocytosis is blocked at 4 °C [35, 36], we hypothesized that NK-EV uptake was occurring via an endocytic process. Indeed, when we incubated K562 cells with PKH67-labeled NK-EVs with ethylisopropylamiloride (EIPA), a specific macropinocytosis inhibitor, the percentage of cells that took up NK-EVs was significantly reduced (Fig. 2D). We also tested Jurkat cells and found that the percentage of these cells that took up NK-EVs was also significantly reduced by treatment with EIPA (Supplementary Fig. 2). These results indicate that NK-EVs are incorporated into target cells via macropinocytosis.

### Treatment with IL-15 + IL-21 enhances the cytotoxic activity of NK-EVs

**Fig. 3:**
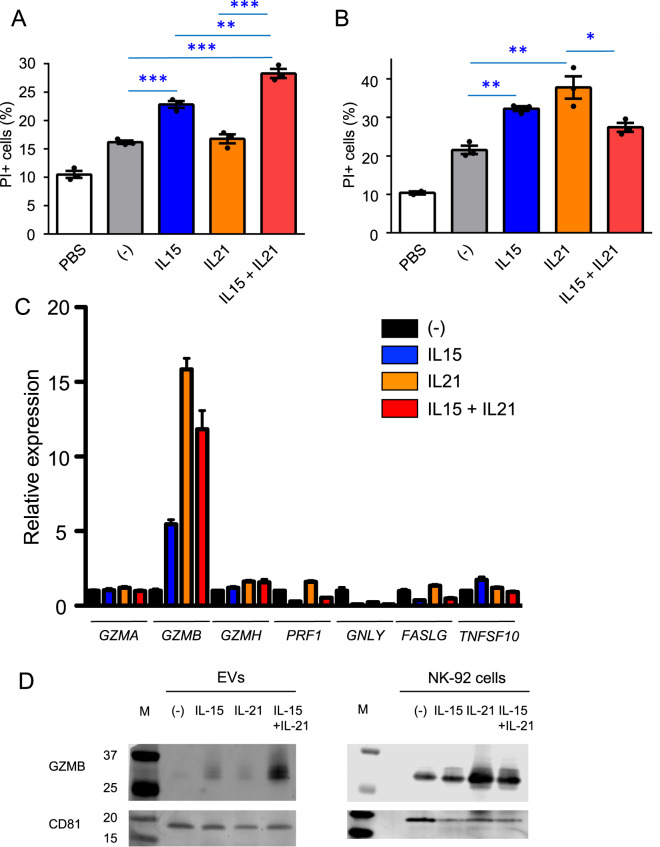
Stimulation with IL-15 plus IL-21 enhances cytotoxic activity of NK-EVs. **A** CFSE-labeled K562 cells (2 × 10^4^) were cultured with PBS or 3 μg of NK-EVs from cells cultured with no cytokine, IL-15, IL-21, or IL-15 + IL-21 for 12 h. The graph represents the means + SEM of percentages of CFSE^+^/PI^+^ K562 cells. Data are shown as means + SEM of three samples. **B** CFSE-labeled K562 cells (5 × 10^3^) were co-cultured with NK-92 cells (5 × 10^4^) cultured with no cytokine, IL-15, IL-21, or IL-15 + IL-21 for 4 h. The graph represents the means + SEM of percentages of CFSE^+^/PI^+^ K562 cells. Data are shown as means + SEM of three samples. **C** qRT-PCR was performed with RNA from NK-92 cells cultured with no cytokine, IL-15, IL-21, or IL-15 + IL-21 for 5 days. Data are shown as means + SEM of three samples. **D** Lysates of NK-EVs and NK-92 cells cultured with no cytokine, IL-15, IL-21, or IL-15 + IL-21 for 5 days were immunoblotted with anti-granzyme B and CD81 (loading control) Ab. M, protein marker. All data shown are representative of three independent experiments. *P < 0.05; **P < 0.01; ***P < 0.001.

We next investigated the effect of the cytokines, IL-15 and IL-21, on the cytotoxic activity of NK-EVs. IL-15 is well known to activate NK cells and enhance their cytotoxic activity, and IL-21 is another γ_c_ family cytokine known to affect the cytolytic activity and the survival of these cells [21–23, 37]. NK-92 cells were cultured with no cytokine, IL-15, IL-21, or IL-15 + IL-21 for 5 days, EVs were isolated from the culture supernatants, and then they, along with PBS as a control, were analyzed for cytotoxic activity against K562 cells. EVs from untreated NK-92 cells as compared to PBS increased killing of K562 cells (Fig. 3A). IL-15 further enhanced killing whereas IL-21 did not, but the combination of IL-15 + IL-21 showed greater cytotoxic activity (Fig. 3A). We also examined the cytotoxic activity mediated by NK-92 cells cultured with no cytokine, IL-15, IL-21, or IL-15 + IL-21 against K562 target cells in co-culture experiments and unexpectedly found that the trend was different, with IL-21 stimulation inducing the highest level of killing (Fig. 3B). To further investigate the effect of cytokine stimulation of these cells, we next used quantitative RT-PCR (qRT-PCR) to examine expression of mRNAs in NK-92 cells encoding proteins related to cytotoxic activity, including granzyme A (*GZMA*), granzyme B (*GZMB*), granzyme H (*GZMH*), perforin (*PRF1*), granulysin (*GNLY*), Fas ligand (*FASLG*), and TRAIL (*TNFSF10*). Of these, only *GZMB* was highly induced by cytokine stimulation (Fig. 3C). The trend of the relative expression levels of *GZMB* was consistent with the trend of cytotoxic activity mediated by NK-92 cells: IL-21 induce more cytotoxic activity by NK-92 cells and more *GZMB* expression as compared to other cytokine conditions (Fig. 3B, C). To assess the levels of GZMB protein, we next performed western blotting in both NK-EVs and NK-92 cells not stimulated or stimulated with IL-15, IL-21, IL-15 + IL-21 (Fig. 3D). Interestingly, the expression levels of GZMB were different between NK-EVs and NK-92 cells. GZMB was reproducibly more highly enriched in NK-EVs from cells treated with IL-15 + IL-21 as compared to other cytokine conditions, consistent with the enhanced cytotoxic activity of NK-EVs by IL-15 + IL-21. In contrast, GZMB was enriched in NK-92 cells treated with IL-21 as compared to other cytokine conditions, consistent with the enhanced cytotoxic activity mediated by NK-92 cells (Fig. 3B) and enhanced *GZMB* expression by IL-21 (Fig. 3C). Because GZMB is a major cytotoxic molecule that can induce apoptosis of target cells [38–40], these results suggested that it was a rational candidate for explaining the enhanced cytotoxic activity of NK-EVs from cells treated with IL-15 + IL-21.

### Treatment with cytokines alters the miRNA profiles in NK-EVs

**Fig. 4:**
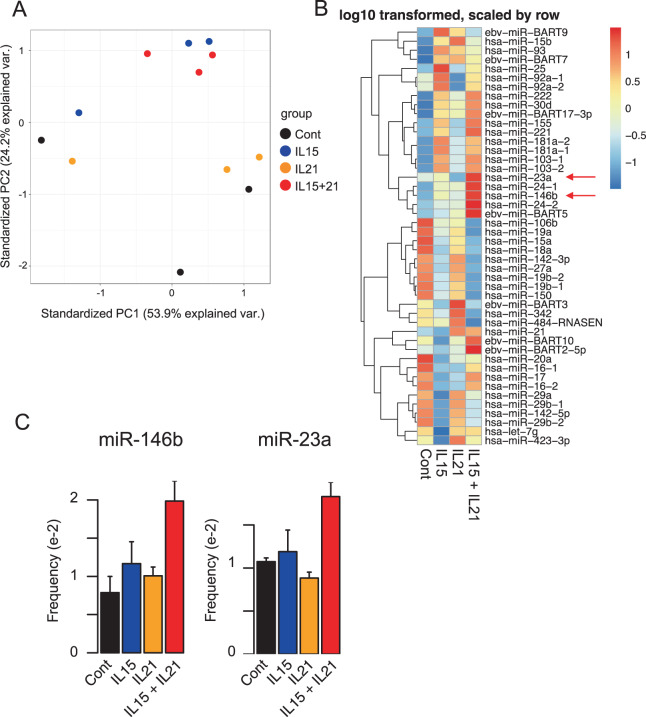
Distinct expression patterns of miRNAs were exhibited in response to different cytokine stimulations. **A** Principal component analysis of small RNA-seq data from NK-EVs and NK-92 cells stimulated with IL-15, IL-21, IL-15 + IL-21, or without any cytokines. Small RNA-seq were performed in triplicate. **B** Heat map of differentially expressed miRNAs in NK-EVs derived from NK-92 cells stimulated with IL-15, IL-21, IL-15 + IL-21, or without any cytokines. All microRNAs with frequency greater than 0.005 (accounting for more than 0.5% of total sequenced reads) were selected and shown. **C** The frequency of miR-146b and miR-23a in NK-EVs stimulated with IL-15, IL-21, IL-15 + IL-21, or without any cytokines. Data are shown as means + SEM of triplicates.

Because miRNAs in EVs are known to mediate effects on target cells, we next performed small RNA-seq to examine the expression profiles of miRNAs in NK-EVs derived from NK-92 cells treated with no cytokine, IL-15, IL-21, or IL-15 + IL-21. The principal component analysis (PCA) plot and the heat map revealed that cytokine stimulation altered the miRNA profiles in NK-EVs (Fig. 4A, B and Supplementary Table 1). In addition, NK-EVs from NK-92 cells treated with IL-15 and IL-15 + IL-21 showed more similar expression patterns than NK-EVs from NK-92 cells treated with either no cytokine or IL-21 (Fig. 4A).

Although many miRNAs were not detected in NK-EVs, several miRNAs, including miR-146b and miR-23a showed significant enrichment in NK-EVs treated with IL-15 + IL-21 as compared to other conditions (Fig. 4B, C). The target genes of these miRNAs, listed from miRTarBase (http://mirtarbase.mbc.nctu.edu.tw/php/index.php), include genes that have oncogenic roles in cancer (Supplementary Table 2). Among them, several genes that promote tumorigenesis in cancer cells, including STAT3, HES1, and genes related to NF-κB signaling could be targeted by miR-23a and miR-146b. Taken together, small RNA-seq data revealed that expression profiles of miRNAs in NK-EVs were altered by cytokine stimulation, consistent with the possibility that miRNAs enriched by cytokine stimulation might contribute to cytotoxic activity of NK-EVs by regulating tumorigenesis in target cells.

### Treatment with cytokines alter the protein profiles in NK-EVs

**Fig. 5:**
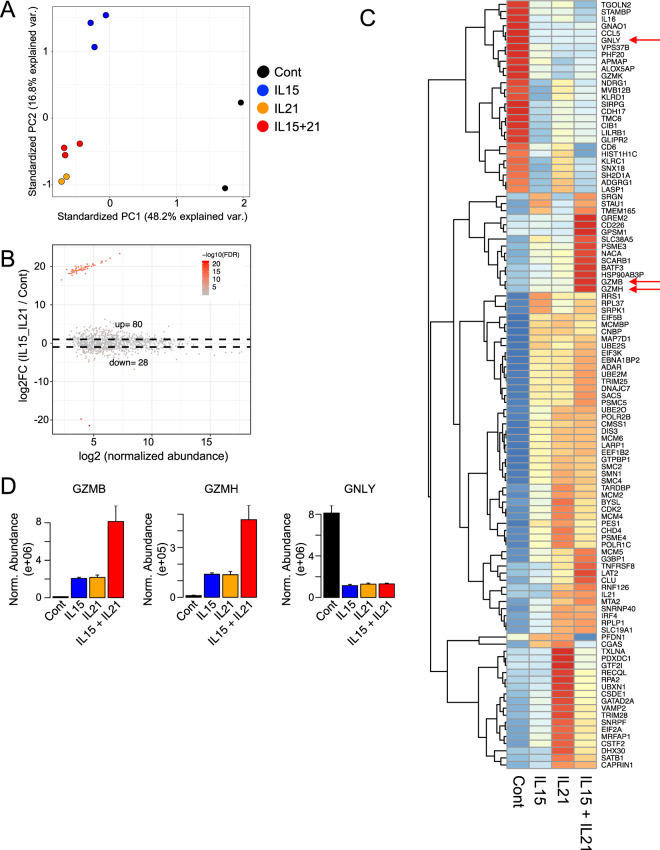
Mass spectrometry revealed protein profiles in NK-EVs. **A** Principal component analysis of mass spectrometry data of EVs derived from NK-92 cells stimulated with IL-15, IL-21, IL-15 + IL-21, or without any cytokines. Duplicate samples were analyzed for no cytokine (control) and IL-21 and triplicate samples for IL-15 and IL-15 + IL-21. **B** MA plot (plot of log-fold changes (*M* values) versus mean normalized expression (*A* values) of differentially expressed proteins in NK-EVs derived from NK-92 cells stimulated with IL-15 + IL-21 or without any cytokines. **C** Heat map of differentially expressed proteins based on FC > 2 and FDR < 0.05 in NK-EVs stimulated with IL-15, IL-21, IL-15 + IL-21, or without any cytokines. **D** The abundance of GZMB, GZMH, and GNLY in NK-EVs derived from NK-92 cells. Data are shown as means + SEM of triplicate or duplicate independent samples analyzed by mass spectrometry.

We next used mass spectrometry to investigate the protein profiles of EVs from NK-92 cells treated with no cytokine, IL-15, IL-21, or IL-15 + IL-21. PCA revealed that cytokine stimulation altered the protein profiles of NK-EVs as compared to the no cytokine control condition (Fig. 5A and Supplementary Table 3). NK-EVs from cells stimulated with IL-21 and with IL-15 + IL-21 showed the most similar protein profiles, whereas NK-EVs from cells treated with IL-15 showed more of a difference (Fig. 5A). As expected, we observed both upregulated and down-regulated proteins when we compared NK-EVs with cytokine stimulation compared to control NK-EVs (Fig. 5B, C). We focused on NK-EVs stimulated with IL-15 + IL-21 because those NK-EVs showed the highest cytotoxic capacity in Fig. 3A. Approximately 80 proteins were enriched in NK-EVs from cells stimulated with cytokines as compared to control cells, and 28 proteins were down-regulated (Fig. 5B, C and Supplementary Fig. 3).

We also performed a GO analysis with the 80 proteins enriched by IL-15 + IL-21 stimulation to investigate the enriched pathway and function (Fig. 5C and Table 1). Interestingly, proteins related to DNA replication, cell cycle, ribonucleic protein complex, metabolic and catabolic pathway were enriched in NK-EVs derived from cells stimulated with IL-15 + IL-21, which suggests these proteins might affect cell processes in target cells (Table 1). Among cytotoxic molecules that directly induce apoptosis in target cells, only GZMB and GZMH were cooperatively induced by IL-15 + IL-21 (see red arrows in Fig. 5C, D and Table 1). Another cytotoxic molecule, GNLY, was not similarly induced (red arrow Fig. 5C, D), so not all cytotoxic molecules were enriched in NK-EVs by treatment with IL-15 + IL-21. These results indicate that the protein profiles in NK-EVs are vastly altered by different cytokine condition, which is assumed to affect the function of NK-EVs.

**Table 1:** Gene ontology analysis of upregulated proteins in EVs derived from NK-92 cells stimulated with IL-15 + IL-21.

Enrichment FDR	Genes in list	Total genes	Functional category	Genes
8.58E−07	8	93	DNA duplex unwinding	RECQL RPA2 MCM2 MCM6 MCM4 CHD4 DHX30 G3BP1
9.74E−07	8	103	DNA geometric change	RECQL RPA2 MCM2 MCM6 MCM4 CHD4 DHX30 G3BP1
2.94E−06	13	503	Ribonucleoprotein complex biogenesis	SRPK1 PES1 BYSL EBNA1BP2 RRS1 SNRPF ADAR SMN1 DIS3 DHX30 EIF3K EIF2A G3BP1
2.94E−06	11	325	DNA conformation change	RECQL PSME4 RPA2 SMC4 MCM2 MCM6 MCM4 CHD4 DHX30 G3BP1 SRPK1
4.44E−06	17	986	MRNA metabolic process	SRPK1 CSTF2 TARDBP SNRNP40 SNRPF SMN1 GTPBP1 ADAR CSDE1 LARP1 POLR2B PSME4 DIS3 PSMC5 PSME3 RPLP1 RPL37
5.85E−06	4	11	DNA unwinding involved in DNA replication	RPA2 MCM2 MCM6 MCM4
2.91E−05	18	1293	Chromosome organization	CHD4 MTA2 RECQL PSME4 RPA2 MCMBP SMC4 CDK2 MCM2 MCM6 MCM4 TRIM28 DHX30 IRF4 G3BP1 SATB1 RRS1 SRPK1
2.96E−05	5	41	DNA replication initiation	MCM2 MCM6 MCM5 MCM4 CDK2
3.24E−05	9	270	Ribonucleoprotein complex assembly	SRPK1 SNRPF ADAR SMN1 EIF3K DHX30 EIF2A G3BP1 RRS1
4.46E−05	9	284	Ribonucleoprotein complex subunit organization	SRPK1 SNRPF ADAR SMN1 EIF3K DHX30 EIF2A G3BP1 RRS1
5.50E−05	3	6	Granzyme-mediated apoptotic signaling pathway	GZMH GZMB SRGN
5.50E−05	22	2054	Negative regulation of gene expression	TARDBP TRIM28 MTA2 GATAD2A CDK2 BATF3 ADAR CNBP PSMC5 GTPBP1 CHD4 CAPRIN1 SATB1 CSDE1 LARP1 NACA POLR2B PSME4 DIS3 PSME3 RPLP1 RPL37
6.89E−05	7	156	DNA-dependent DNA replication	RPA2 MCMBP MCM2 MCM6 MCM5 MCM4 CDK2
0.000110948	8	244	Regulation of viral process	SRPK1 TARDBP TRIM25 TRIM28 ADAR STAU1 LARP1 POLR2B
0.00011331	10	431	MRNA catabolic process	TARDBP GTPBP1 CSDE1 LARP1 PSME4 DIS3 PSMC5 PSME3 RPLP1 RPL37
0.000121967	12	662	Posttranscriptional regulation of gene expression	TARDBP EIF5B ADAR CAPRIN1 EIF2A LARP1 EIF3K POLR2B PSME4 DIS3 PSMC5 PSME3
0.000193085	10	465	RNA catabolic process	DIS3 TARDBP GTPBP1 CSDE1 LARP1 PSME4 PSMC5 PSME3 RPLP1 RPL37
0.000193085	8	272	Regulation of symbiosis, encompassing mutualism through parasitism	SRPK1 TARDBP TRIM25 TRIM28 ADAR STAU1 LARP1 POLR2B
0.000345667	4	37	Positive regulation of viral genome replication	SRPK1 ADAR STAU1 LARP1
0.000370357	34	4923	Regulation of gene expression	PSMC5 SRPK1 TARDBP TNFRSF8 TRIM28 MTA2 GATAD2A CDK2 BATF3 IRF4 EIF5B ADAR CNBP UBE2O GTPBP1 CHD4 CAPRIN1 EIF2A LARP1 EIF3K GREM2 SATB1 GTF2I CSDE1 CLU TRIM25 NACA POLR2B PSME4 DIS3 PSME3 RPLP1 RPL37 POLR1C
0.000452642	7	223	Regulation of mRNA catabolic process	TARDBP GTPBP1 LARP1 PSME4 DIS3 PSMC5 PSME3
0.00064703	14	1093	DNA metabolic process	RECQL RPA2 MCMBP TRIM28 PSME4 MCM2 MCM6 MCM5 MCM4 CDK2 MTA2 POLR2B TRIM25 GATAD2A
0.000656406	13	951	Viral process	SRPK1 TARDBP TRIM25 TRIM28 ADAR SCARB1 STAU1 UBXN1 SATB1 LARP1 POLR2B RPLP1 RPL37
0.000777493	15	1276	Cellular macromolecule catabolic process	PSME4 DIS3 UBE2S CLU TARDBP PSME3 PSMC5 UBXN1 GTPBP1 CSDE1 TRIM25 LARP1 CDK2 RPLP1 RPL37
0.000854984	5	101	Anaphase-promoting complex-dependent catabolic process	UBE2S PSME4 PSMC5 CDK2 PSME3
0.000879296	8	358	Regulation of mRNA metabolic process	SRPK1 TARDBP GTPBP1 LARP1 PSME4 DIS3 PSMC5 PSME3
0.000879296	6	174	Regulation of viral life cycle	SRPK1 TRIM25 TRIM28 ADAR STAU1 LARP1
0.000879296	7	260	G1/S transition of mitotic cell cycle	CDK2 PSME3 RPA2 MCM2 MCM6 MCM5 MCM4
0.000879296	24	2980	Negative regulation of macromolecule metabolic process	TARDBP TRIM28 MTA2 GATAD2A CLU CDK2 BATF3 ADAR UBXN1 CNBP PSMC5 GTPBP1 CHD4 CAPRIN1 SATB1 CSDE1 LARP1 NACA POLR2B PSME4 DIS3 PSME3 RPLP1 RPL37
0.000879296	5	103	Regulation of viral genome replication	SRPK1 ADAR TRIM28 STAU1 LARP1

### GZMB did not contribute to the cytolytic activity of NK-EVs

**Fig. 6:**
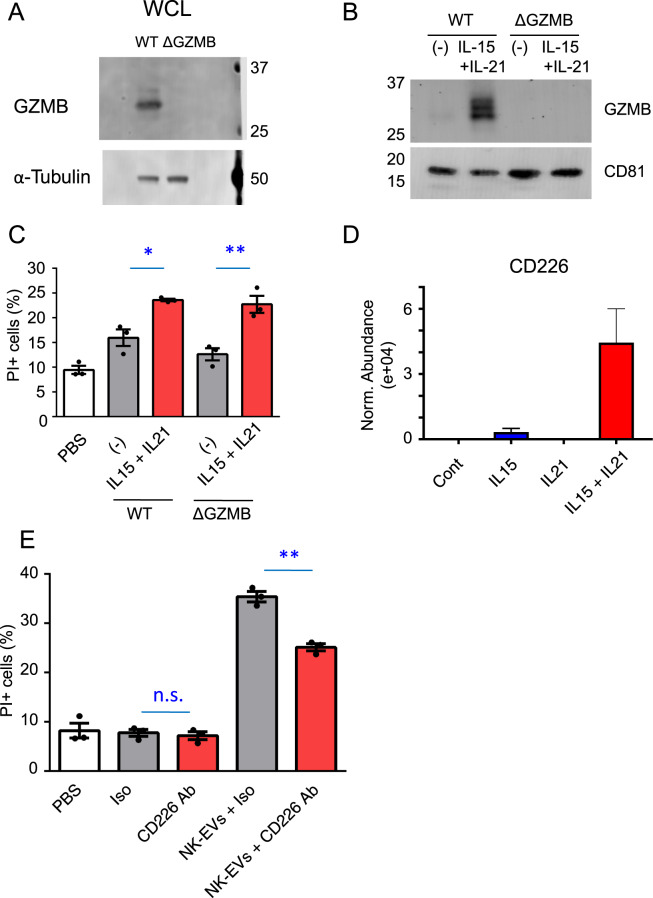
Blocking of CD226 inhibits the cytolytic activity of NK-EVs. **A** Whole-cell lysates (WCL) of NK-92 WT and ΔGZMB cells were immunoblotted with antibodies to GZMB and α-tubulin (loading control). **B** Lysates of NK-EVs from WT and ΔGZMB NK-92 cells stimulated with IL-15 + IL-21 or without any cytokine were immunoblotted with antibodies to granzyme B and CD81 (loading control). **C** CFSE-labeled K562 cells (2 × 10^4^) were cultured with 3 μg of NK-EVs from WT and ΔGZMB NK-92 cells stimulated with IL-15 + IL-21 or without any cytokine for 12 h. The graph represents the means + SEM of percentages of CFSE^+^/PI^+^ K562 cells. Data are shown as means + SEM of three samples and representative of three independent experiments. **D** The abundance of CD226 in NK-EVs derived from NK-92 cells. The data were from mass spectrometry analyses. **E** CFSE-labeled K562 cells (2 × 10^4^) were cultured with 3 μg of NK-EVs from NK-92 cells stimulated with IL-15 + IL-21 for 12 h. NK-EVs were preincubated with isotype control or anti-CD226 antibodies for 30 min at 37 °C and then incubated with K562 cells. n.s. not significant, The graph represents the means + SEM of percentages of CFSE^+^/ PI^+^ K562 cells. Data are shown as means + SEM of three samples and representative of three independent experiments. *P < 0.05; **P < 0.01.

To investigate the function of GZMB in NK-EVs, we generated *GZMB*-deficient (ΔGZMB) cells by using CRISPR-Cas9 technology with the gRNA targeting the exon 4 of *GZMB* (Supplementary Fig. 4A). This resulted in loss of a nucleotide, causing a frameshift and premature stop codon in the exon 4 (Supplementary Fig. 4B), which caused the loss of expression of *GZMB* mRNA, as confirmed by primer pairs that bind to exon 4/exon 5, downstream of the mutated site (Supplementary Fig. 4C). No mRNA was detected even 5′ to the mutation by primer pairs that bind to exon 2/exon 3, upstream of the mutated site (Supplementary Fig. 4C), suggesting that the mutation caused instability/degradation of *GZMB* mRNA [41, 42]. Correspondingly, no GZMB protein was detected either in the cells (Fig. 6A and Supplementary Fig. 4D) or in the NK-EVs from ΔGZMB cells (Fig. 6B and Supplementary Fig. 4E). Because *GZMH* shares high sequence identity of mRNA (~80%) with *GZMB*, we assessed whether *GZMH* was also disrupted in ΔGZMB cells by the same guide RNA used for targeting *GZMB* (Supplementary Fig. 5A–D). Indeed, the gRNA resulted in the loss of two nucleotides, causing a frameshift and premature stop codon in the exon 4 of *GZMH* (Supplementary Fig. 5B), profoundly decreased *GZMH* mRNA assessed with primer pairs for either exon 4/exon 5 or exon 1/exon 2, upstream of the mutated site (Supplementary Fig. 5C), and no detectable GZMH protein (Supplementary Fig. 5D).

Surprisingly, however, the cytotoxic activity of NK-EVs from ΔGZMB cells was still enhanced by treatment with IL-15 + IL-21 (Fig. 6C), indicating that neither GZMB nor GZMH was substantially involved in enhancing cytotoxic activity of NK-EVs by treatment with these cytokines and that other mechanism(s) are involved in enhancing cytotoxic activity of NK-EVs by treatment of NK-92 cells with IL-15 + IL-21.

### CD226 on NK-EVs contributes to the cytolytic activity

Careful examination of the data in Fig. 5C reveals that a range of proteins besides GZMB and GZMH are also cooperatively induced by the combination of IL-15 + IL-21 (e.g., GREM2, CD226, GPSM1, SLC38A5, PSME3, NACA, SCARB1, BATF3, and HSP90AB3P), providing a range of other molecules that potentially might directly or indirectly influence the enhanced killing by NK-EVs. Among those proteins, we next focused on CD226, because soluble recombinant CD226 and also CD226 on NK-EVs have cytolytic activity against tumor cells as reported previously [43, 44]. As shown in Fig. 6D, CD226 was significantly enriched on NK-EVs by IL-15 + IL-21 stimulation. We then examined cytotoxic assay with anti-CD226-blocking antibodies and found that cytolytic activity of NK-EVs was inhibited by blocking of CD226. These results suggest that enhanced cytolytic activity of NK-EVs by IL-15 + IL-21 stimulation is partially explained by enriched CD226 on NK-EVs.

## Discussion

Exosomes or EVs contain various cellular components, including proteins, DNAs, mRNAs, and miRNAs and play roles as mediators for intercellular communication [8, 12]. Since EVs derived from cancer cells can be identified in body fluids including blood and urine, analysis of EVs may have diagnostic value in cancer [45, 46]. Moreover, previous studies showed that EVs are quite stable and can selectively target certain organs or cell types [10, 11, 47, 48]. Therefore, EVs can potentially be utilized clinically, including for drug delivery, vaccination, and tissue repair [49–51].

In this study, we found that NK-EVs are incorporated into target cells via macropinocytosis, one of the endocytic pathways. To our knowledge, this is the first report to identify the pathway of NK-EVs-uptake by recipient cells. Lugini et al. [5] mentioned NK-EVs isolated from plasma of healthy donors have cytolytic activity against cancer cells but not normal resting PBMC cells and proposed NK-EVs have function for immune surveillance in our body. However, the mechanism as to how NK-EVs distinguish between tumor cells and normal cells has remained unknown. The macropinocytosis pathway is known to be an important nutrient-scavenging pathway in numerous cancer types, including pancreatic, lung, prostate, and bladder cancer [52]. In addition, previous studies reported oncogenic transformation (e.g., oncogenic *RAS* or activated *v-Src* expression), tumor suppressor mutations (e.g., loss of *PTEN*), stimulation with growth factors, such as epidermal growth factor, platelet-derived growth factor, and macrophage colony-stimulating factor enhance macropinocytosis [52]. These results suggested that targeting cancer cells with enhanced macropinocytosis by using EVs including NK-EVs might be an effective strategy, consistent with a previous report [53].

Interestingly, we showed that the cytotoxic activity of EVs derived from NK-92 cells was enhanced by cytokine stimulation, particularly by IL-15 + IL-21. Since IL-15 and IL-21 can activate NK cells [26, 27], it is reasonable that these cytokines can affect the characteristics of EVs derived from NK cells. Interestingly, a cooperative effect on the direct cytotoxic activity of NK-92 cells was not observed, but the treatment of these cells with the combination of IL-15 and IL-21 had cooperative effects on the cytotoxic activity of NK-EVs. The difference in cytotoxic activity seen by treatment with NK-EVs or by direct killing might be explained by the different mechanisms used to kill target cells or by different expression levels of molecules related to cytotoxic activity, but further studies are required to clarify the difference. Although GZMB did not contribute to the enhanced cytotoxic activity of NK-EVs by cytokine stimulation, its expression was different in NK-EVs and whole-cell lysate, which suggests distinctive protein profiles between NK-EVs and NK-92 cells, as previously described [54].

To examine the protein profiles in NK-EVs, we performed mass spectrometry analysis of NK-EVs from NK-92 cells treated with no cytokine, IL-15, IL-21, or IL-15 + IL-21. As expected, the protein profiles in NK-EVs were altered by each cytokine stimulation, which suggested that distinct patterns of components in NK-EVs might have different function on target cells. GO analysis was performed with proteins enriched by cytokine stimulation and those proteins were categorized on DNA replication, cell cycle, ribonucleic protein complex, metabolic and catabolic pathway other than the Granzyme-mediated apoptotic signaling pathway. Those proteins in NK-EVs might be interesting because they have not previously been mentioned related to the function including cytotoxic activity in target cells. However, further studies are required to discover which proteins are important for cytotoxic activity of NK-EVs.

Small RNA-seq also revealed different expression profiles of miRNAs in NK-EVs derived from NK-92 cells treated with no cytokine, IL-15, IL-21, or IL-15 + IL-21. As target genes of each miRNAs are listed in Supplementary Table 3, abundant genes are potentially targeted by miR-23a and miR-146b. Among them, STAT3 and HES1 could be targeted by miR-23a. It is also interesting that many genes related to NF-κB signaling such as IRAK1, TRAF6, CARD10, NFKB1, and TLR4 could be targeted by miR-146b. Whether these or other miRNAs contribute to the cytotoxic activity of NK-EVs requires further studies.

In the present study, we found that cytotoxic activity of NK-EVs was enhanced by treatment with IL-15 + IL-21. Moreover, we found that CD226, which has cytolytic activity against cancer cells, was enriched on NK-EVs by IL-15 + IL-21 stimulation. The addition of blocking antibodies to CD226 reduced the cytotoxic activity of NK-EVs, consistent with a previous report that showed blocking antibodies against CD226-L, CD112 (Nectin2), and CD155 (PVR) could reduce the cytotoxic activity of NK-EVs [44]. CD112 and CD155 are also ligands of TIGIT, which is a checkpoint receptor associated with the antitumor roles on NK and T cells [55]. In addition, these proteins are overexpressed on many types of cancer cells including K562 cells [55, 56]. Therefore, it is reasonable that CD112 and CD155 on cancer cells are targeted by CD226 on NK-EVs, which would contribute to antitumor immune responses. This study is the first report to show the mass spectrometry and small RNA-seq in NK-EVs, which indicates the potential potency and utility of NK-EVs and may inform future studies.

In conclusion, this study revealed novel characteristics of NK-EVs, including the finding that IL-15 + IL-21 stimulation enhances the cytotoxic activity of NK-EVs. Moreover, the NK-EVs-uptake pathway by recipient cells was newly revealed. These results are valuable and informative for developing the strategy for future cancer therapy use of NK-EVs.

## Supplementary information

Suppl. Fig. Legends

Suppl. Figures 1-5

Suppl. Table 1

Suppl. Table 2

Suppl. Table 3

qPCR delta CT values

Original Western Blot Fig. 1

Original Western Blot Fig. 3

Original Western Blot Fig. 6

Original Western Blot Suppl. Fig. 5
