# Supplementary material for: Cytokine-enhanced cytolytic activity of exosomes from NK Cells
Source: Cancer Gene Ther. 2021 Jul 27;29(6):734–49. doi: 10.1038/s41417-021-00352-2 (PMC9209332; doi:10.1038/s41417-021-00352-2)
Supplement: Supplementary file 1 — Suppl. Fig. Legends [file 41417_2021_352_MOESM1_ESM.docx]

**Supplementary Figure Legends**

**Supplementary Fig. 1: Gating strategy for flow cytometric analysis.**

**A-D,** K562, Jurkat, A549, and HeLa cells were treated as described in the legend to Fig. 1C. Cells were gated on FSC-A and SSC-A, which include both live and dead cells. Cells were then further gated on CFSE^+^ cells. Dead cells among CFSE^+^ cells were then measured by PI staining. All data shown are representative of three independent experiments.

**Supplementary Fig. 2: NK-EVs are taken up by Jurkat cells via macropinocytosis.** Jurkat cells were preincubated with DMSO or 75 μM EIPA for 30 minutes. Cells were then incubated with PBS or 3 μg of PKH67-labeled NK-EVs for 3 hours. The percentage of PKH67^+^ Jurkat cells were analyzed by flow cytometry. The graphs represent the means + SEM of the percentage of PKH67^+^ Jurkat cells. Cell culture were performed in triplicate. All data shown are representative of three independent experiments. ***P < 0.001.

**Supplementary Fig. 3: Mass spectrometry analyses of NK-EVs. A,** Venn diagram showing unique and overlapping proteins identified by mass spectrometry analyses. **B,** Volcano plots of differentially expressed proteins in NK-EVs derived from NK-92 cells stimulated with IL-15 + IL-21 or without any cytokines. In panel B, the left and right plots are the same except the top 50 genes are indicated in the plot on the right.

**Supplementary Fig. 4: Generation of ΔGZMB NK-92 cells.**

**A,** The genomic structure of the *GZMB* gene and the sequence of target site of the gRNA are shown. **B,** Sequence of Exon 4 of *GZMB*. The underlined green nucleotide indicates the nucleotide deleted by CRISPR-Cas9 technology, and the three red nucleotides indicate the putative stop codon for *GZM*B in ΔGZMB cells. **C,** qRT-PCR was performed with RNA from NK-92 WT and ΔGZMB cells. The *GZMB* exon 2/exon 3 or *GZMB* exon 4/exon 5 regions were amplified with primer pairs that bind to exon 2/exon 3 or exon 4/exon 5, respectively. Data shown are representative of three independent experiments. Bars indicate the means of triplicates. **D,** Whole Cell Lysates (WCL) of NK-92 WT and ΔGZMB cells were immunoblotted with antibodies to GZMB. **E,** Lysates of NK-EVs from WT and ΔGZMB NK-92 cells stimulated with IL-15 + IL-21 or without any cytokine were immunoblotted with antibodies to GZMB. The whole membrane of Fig. 4E is shown here.

**Supplementary Fig. 5: *GZMH* was disrupted in ΔGZMB NK-92 cells.**

**A,** The sequence of the putative target site in exon 4 of *GZMH* and the sequence of the gRNA are shown. **B,** Sequence of Exon 4 of *GZMH*. The two green nucleotides were deleted by CRISPR-Cas9 technology, and the three red nucleotides indicate the putative stop codon of *GZMH* in ΔGZMB cells. **C,** qRT-PCR was performed with RNA from NK-92 WT and ΔGZMB cells. *GZMH* exon 1/exon 2 or *GZMH* exon 4/exon 5 regions were amplified with primer pairs that bind to Exon 1/Exon 2 or Exon 4/Exon 5, respectively. Data shown are representative of three independent experiments. Bars indicate the means of triplicates. **D,** Whole Cell Lysates (WCL) of NK-92 WT and ΔGZMB cells were immunoblotted with antibodies to GZMH and α-tubulin.
