# Supplementary material for: Cytokine-enhanced cytolytic activity of exosomes from NK Cells
Source: Cancer Gene Ther. 2021 Jul 27;29(6):734–49. doi: 10.1038/s41417-021-00352-2 (PMC9209332; doi:10.1038/s41417-021-00352-2)

sFig. 1

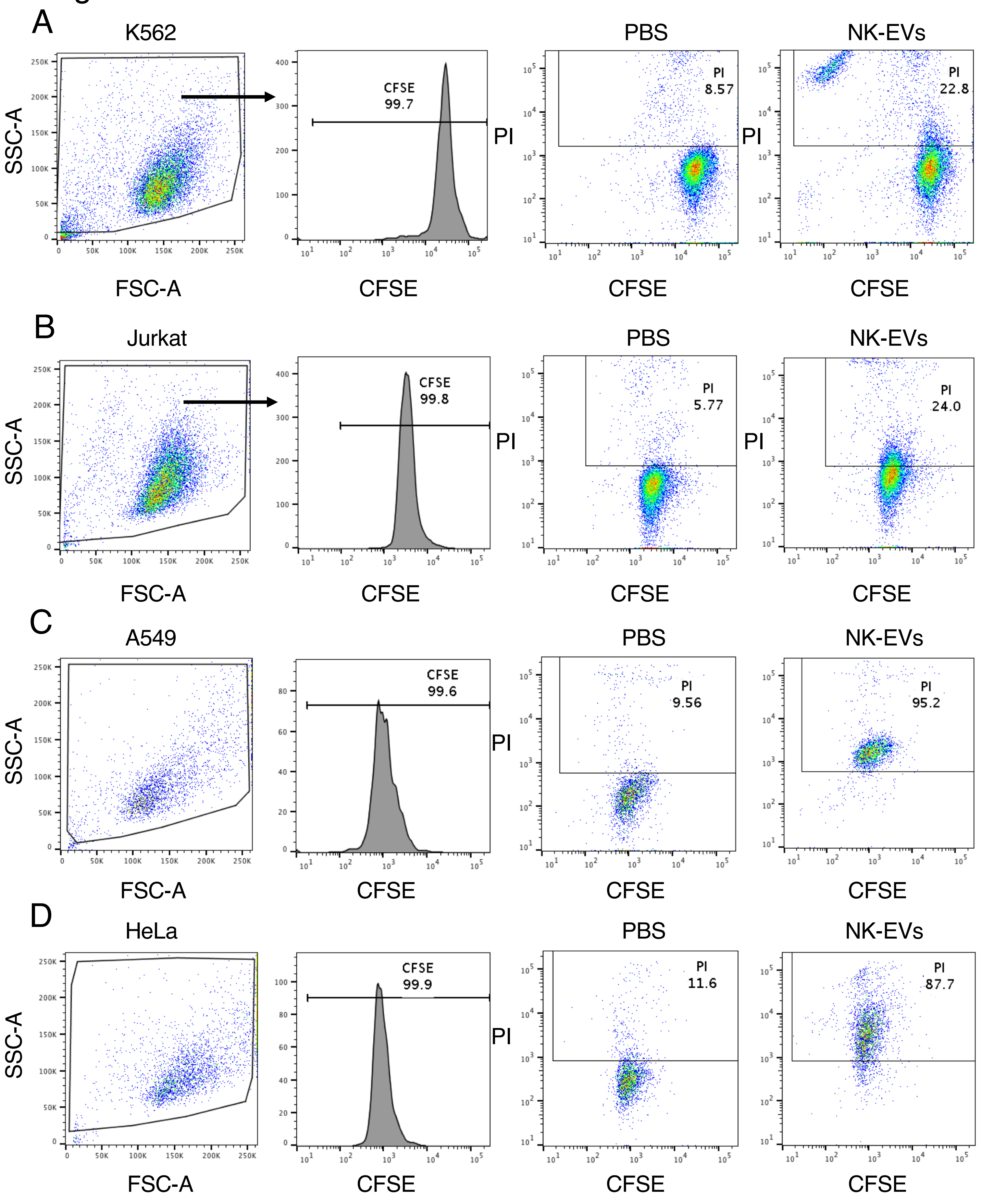
A
K562
SSC-A
FSC-A
CFSE
99.7
PI
PBS
PI
8.57
NK-EVs
PI
22.8
B
Jurkat
CFSE
99.8
PI
5.77
PI
24.0
C
A549
CFSE
99.6
PI
9.56
PI
95.2
D
HeLa
CFSE
99.9
PI
11.6
PI
87.7

sFig. 2

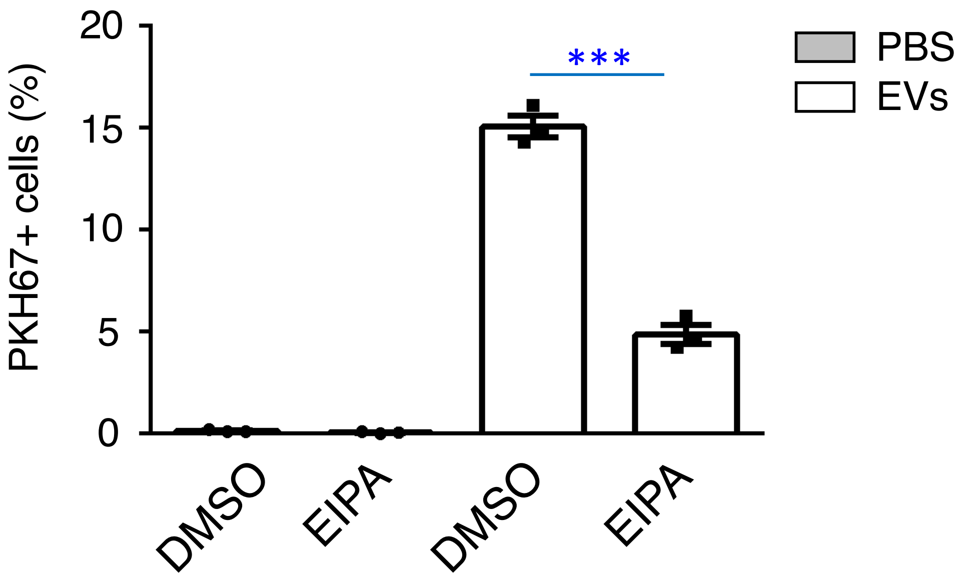

sFig. 3

A

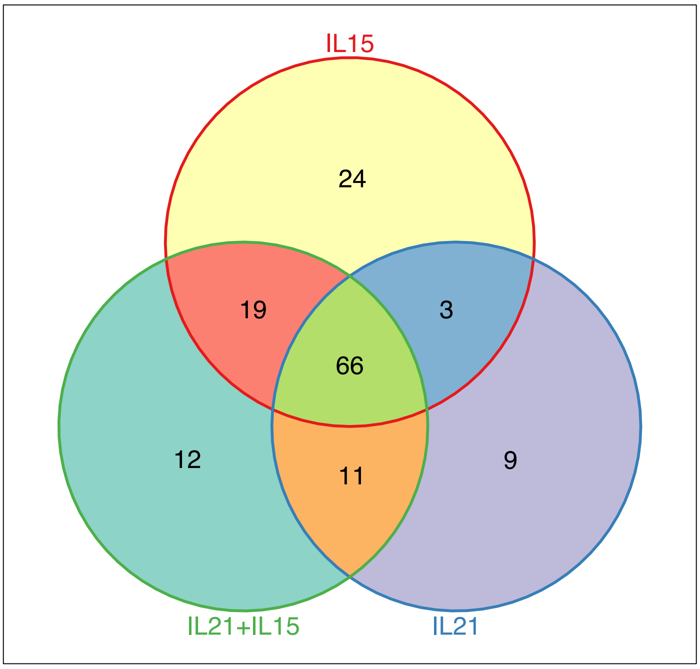

B

Cont vs. IL15 + IL21

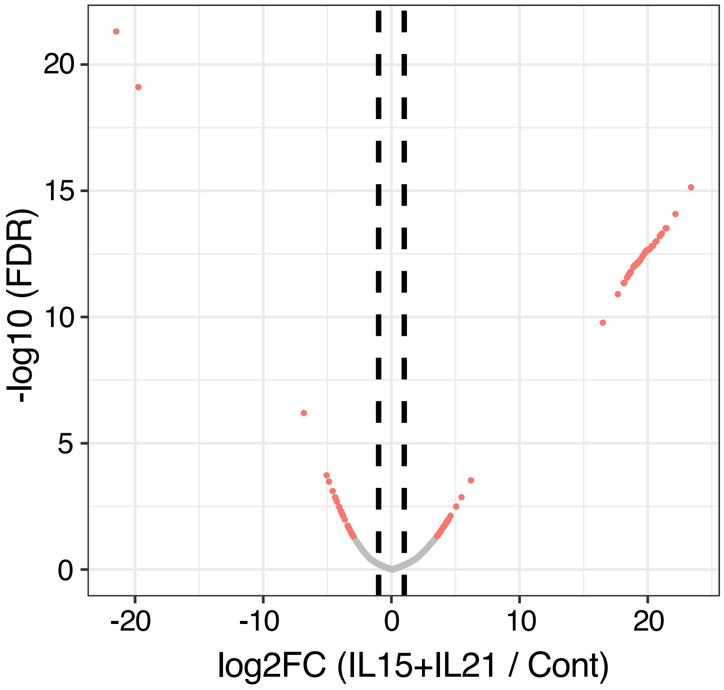

Cont vs. IL15 + IL21, top50 genes

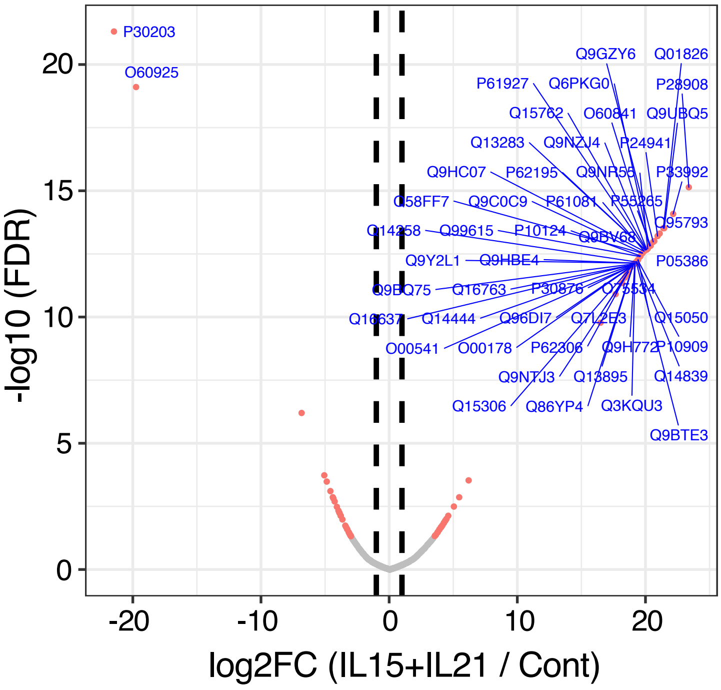

sFig. 4

A

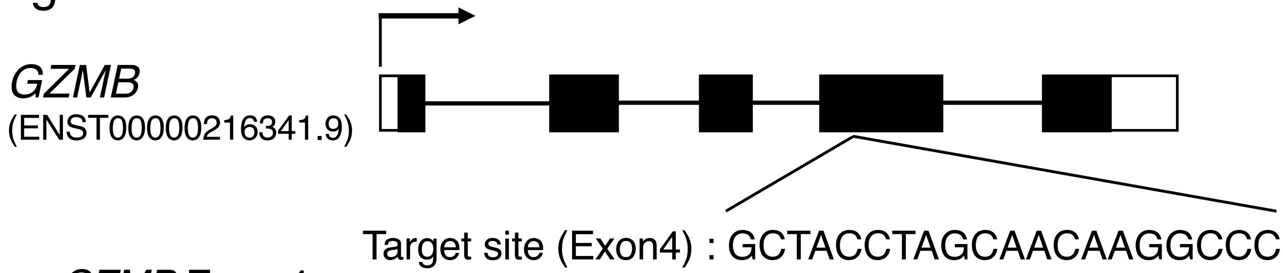

B *GZMB* Exon 4

CTGGAGAGAAAGGCCAAGCGGACCAGAGCTGTGCAGCCCCTCAGGCTACCTAGCAA
CAAGGCCCAGGTGAAGCCAGGGCAGACATGCAGTGTGGCCGGCTGGGGGCAGACG
GCCCCCCTGGGAAAACACTCACACACACTACAAGAGGTGAAGATGACAGTGCAGGAA
GATCGAAAGTGCGAATCTGACTTACGCCATTATTACGACAGTACCATTGAGTTGTGCGT
GGGGGACCCAGAGATTAAAAAGACTTCCTTTAAG

C

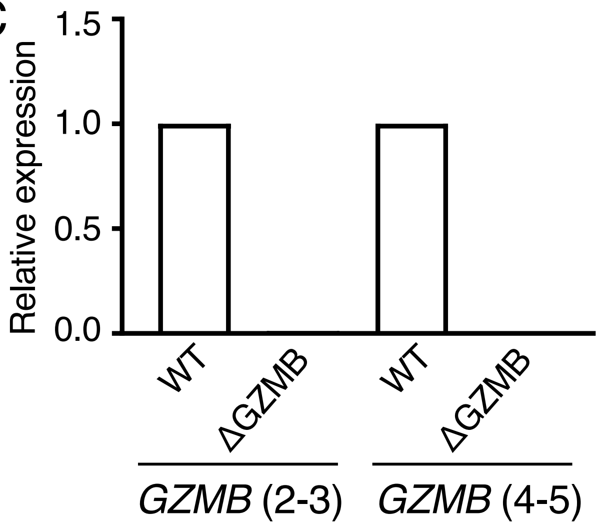

D

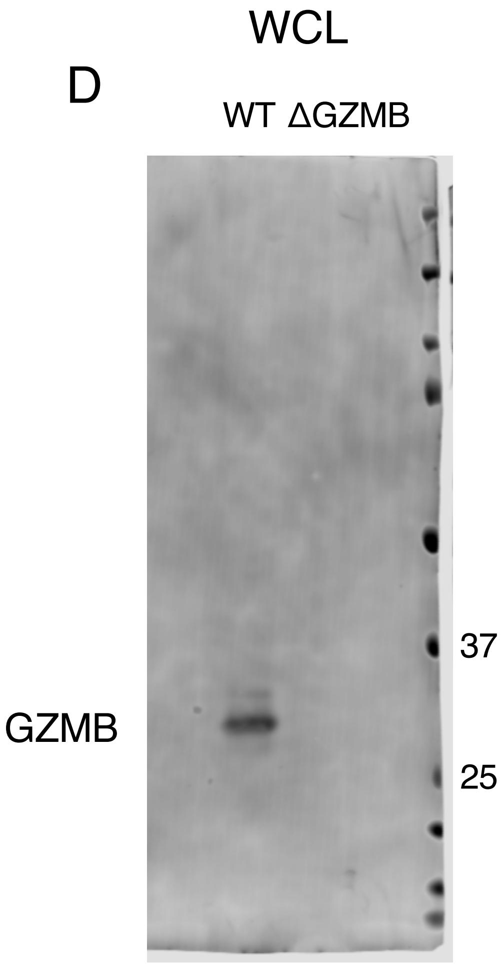

E

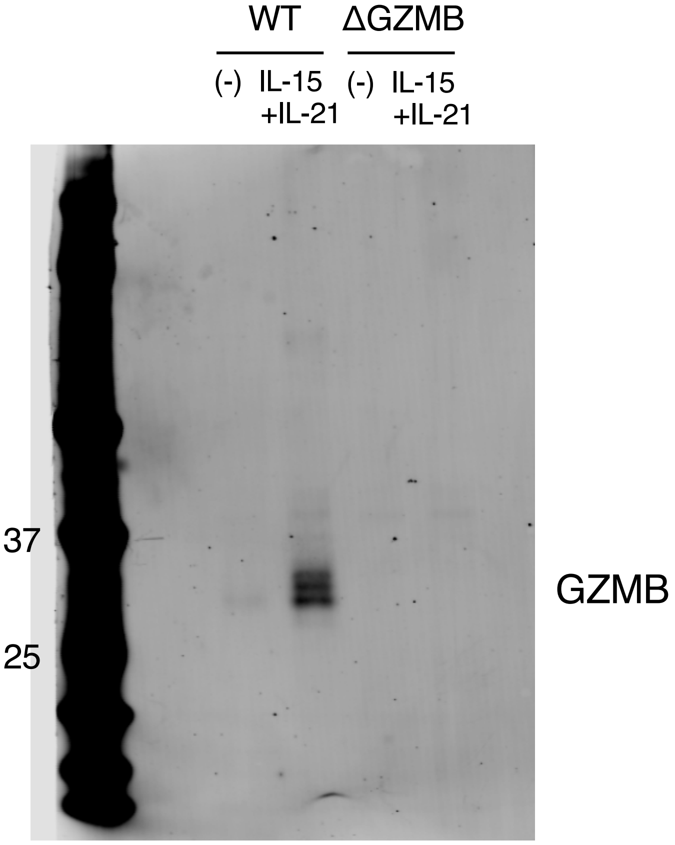

sFig. 5

A

GZMH Exon4 : GCTACCTAGCAGCAAGGCCC
*
gRNA target site : GCTACCTAGCAACAAGGCCC

95% Match

B *GZMH* Exon 4

CTGGAGAGAAAGGCCAAGTGGACCACAGCTGTGCGGCCTCTCAGGCTACCTAGCAGCAAGGCCCAGGTGAAGCCA
GGGCAGCTGTGCAGTGTGGCTGGCTGGGGTTATGTCTCAATGAGCACTTTAGCAACCACACTGCAGGAAGTGTTGC
TGACAGTGCAGAAGGACTGCCAGTGTGAACGTCTCTTCCATGGCAATTACAGCAGAGCCACTGAGATTTGTGTGGG
GGATCCAAAGAAGACACAGACCGGTTTCAAG

C

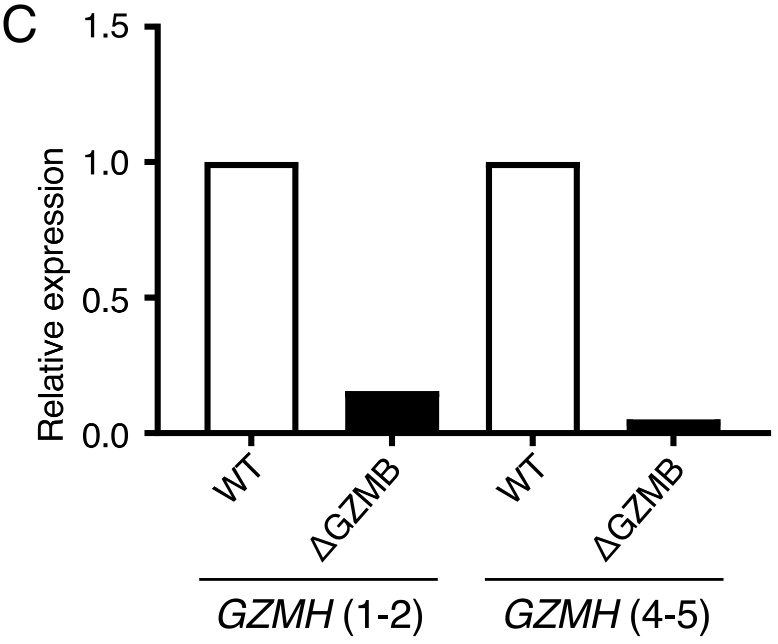

D

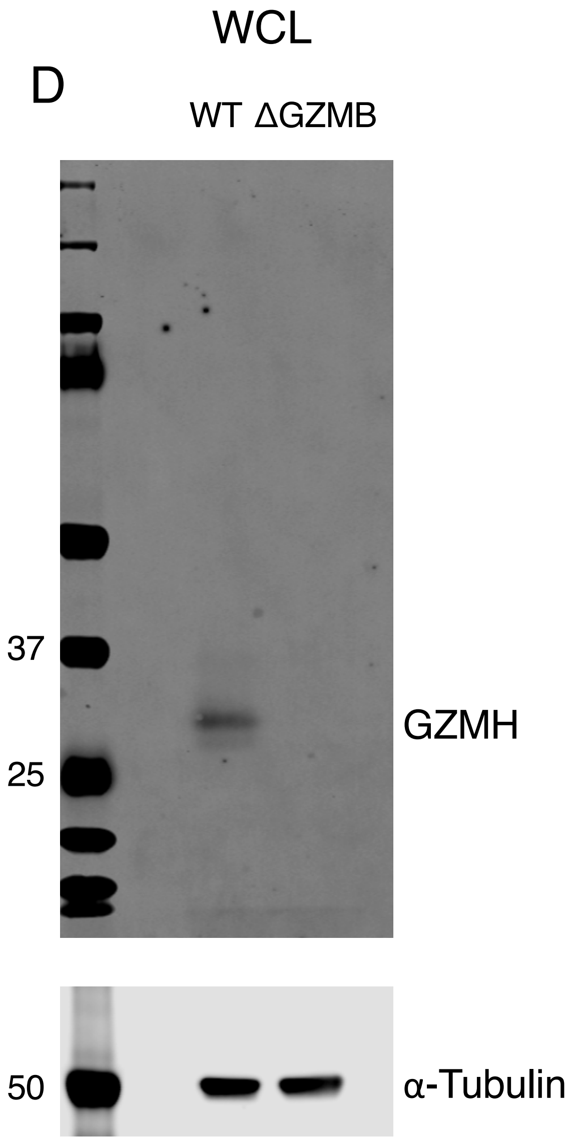

Supplement: Supplementary file 2 — Suppl. Figures 1-5 [file 41417_2021_352_MOESM2_ESM.pdf]
