# Supplementary figures and images for: Cytokine-enhanced cytolytic activity of exosomes from NK Cells
Source: Cancer Gene Ther. 2021 Jul 27;29(6):734–49. doi: 10.1038/s41417-021-00352-2 (PMC9209332; doi:10.1038/s41417-021-00352-2)

## Slide 1
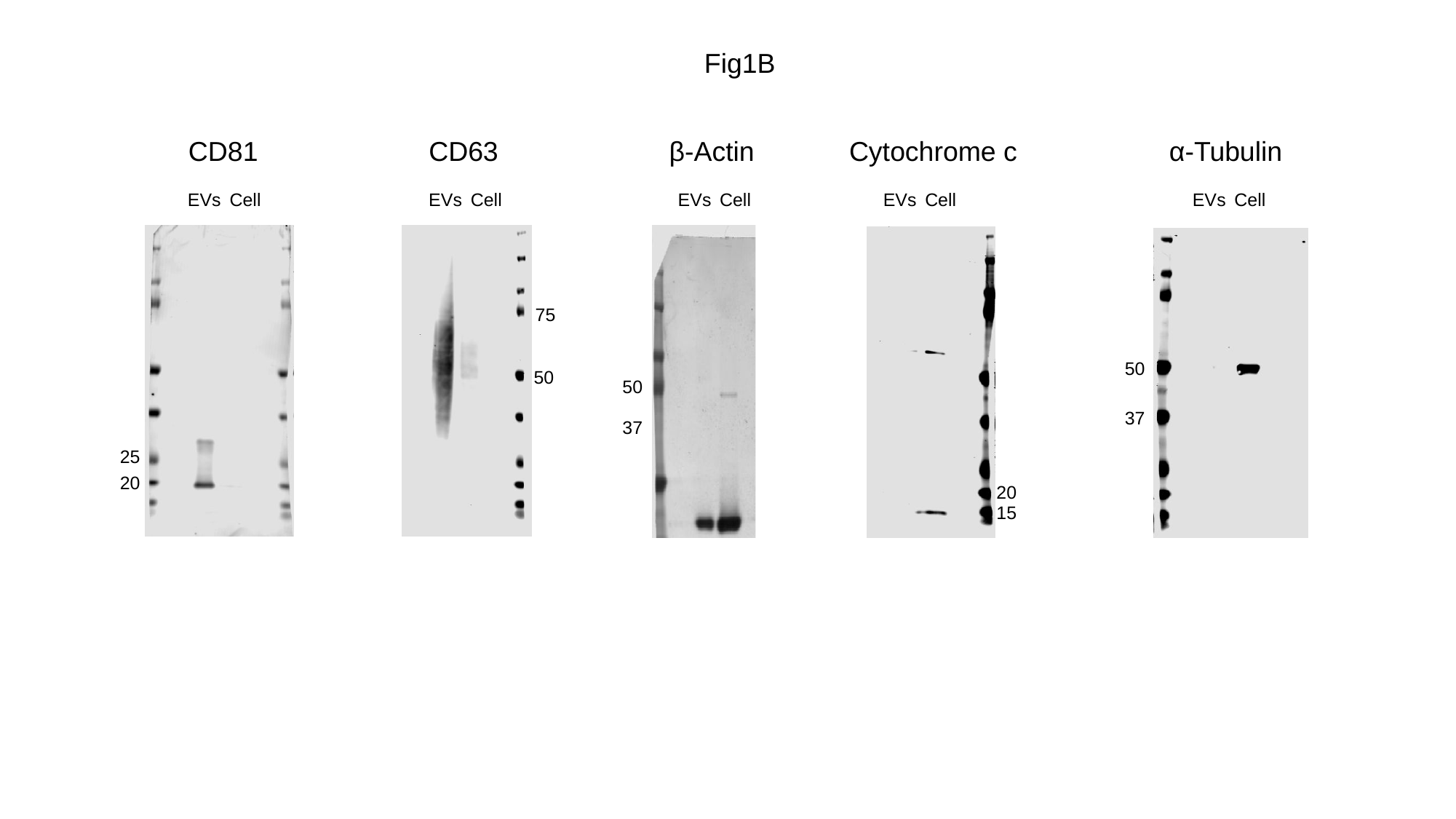

Fig1B
CD81
CD63
β-Actin
Cytochrome c
α-Tubulin
EVs
Cell
EVs
Cell
EVs
Cell
EVs
Cell
EVs
Cell
75
50
50
50
37
37
25
20
20
15

Supplement: Supplementary file 7 — Original Western Blot Fig. 1 [file 41417_2021_352_MOESM7_ESM.pptx]

## Slide 1
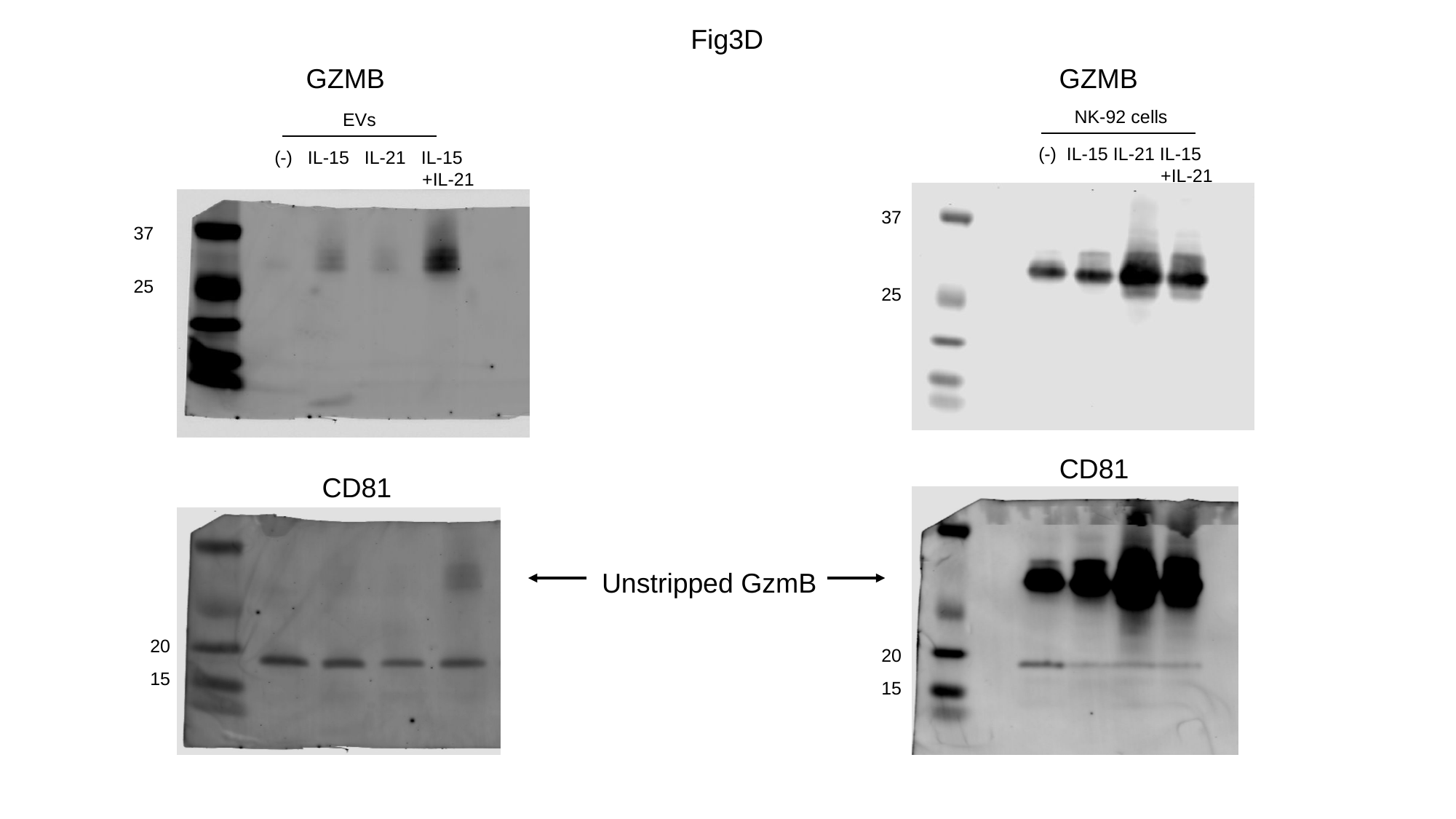

Fig3D
GZMB
GZMB
NK-92 cells
EVs
(-) IL-15 IL-21 IL-15
 +IL-21
(-) IL-15 IL-21 IL-15
 +IL-21
37
37
25
25
CD81
CD81
Unstripped GzmB
20
20
15
15

Supplement: Supplementary file 8 — Original Western Blot Fig. 3 [file 41417_2021_352_MOESM8_ESM.pptx]

## Slide 1
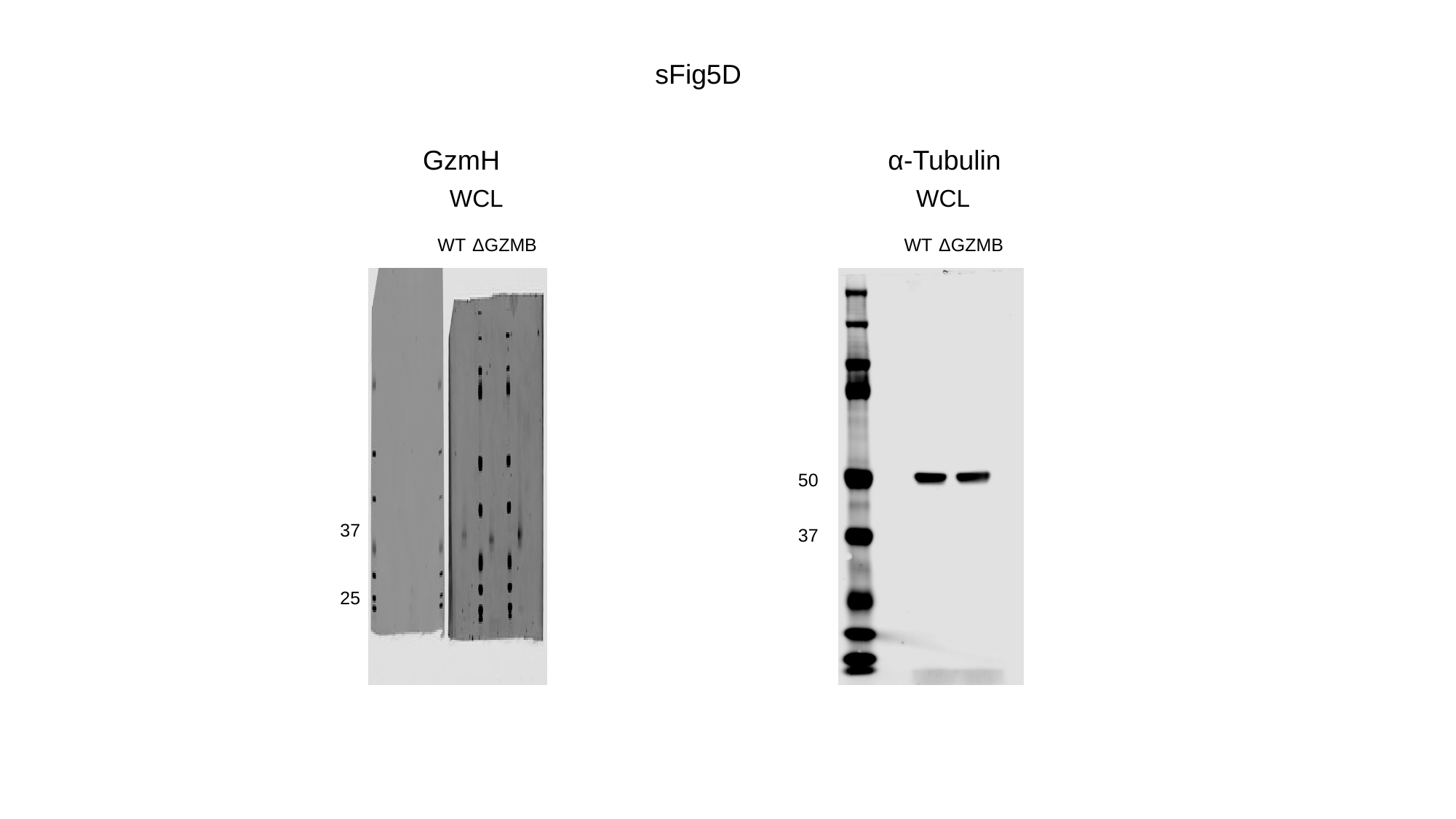

sFig5D
GzmH
α-Tubulin
WCL
WCL
WT
ΔGZMB
WT
ΔGZMB
50
37
37
25

Supplement: Supplementary file 10 — Original Western Blot Suppl. Fig. 5 [file 41417_2021_352_MOESM10_ESM.pptx]
