# Supplementary material for: Cytokine-enhanced cytolytic activity of exosomes from NK Cells
Source: Cancer Gene Ther. 2021 Jul 27;29(6):734–49. doi: 10.1038/s41417-021-00352-2 (PMC9209332; doi:10.1038/s41417-021-00352-2)
Supplement: Supplementary file 9 — Original Western Blot Fig. 6 [file 41417_2021_352_MOESM9_ESM.pptx]

## Slide 1
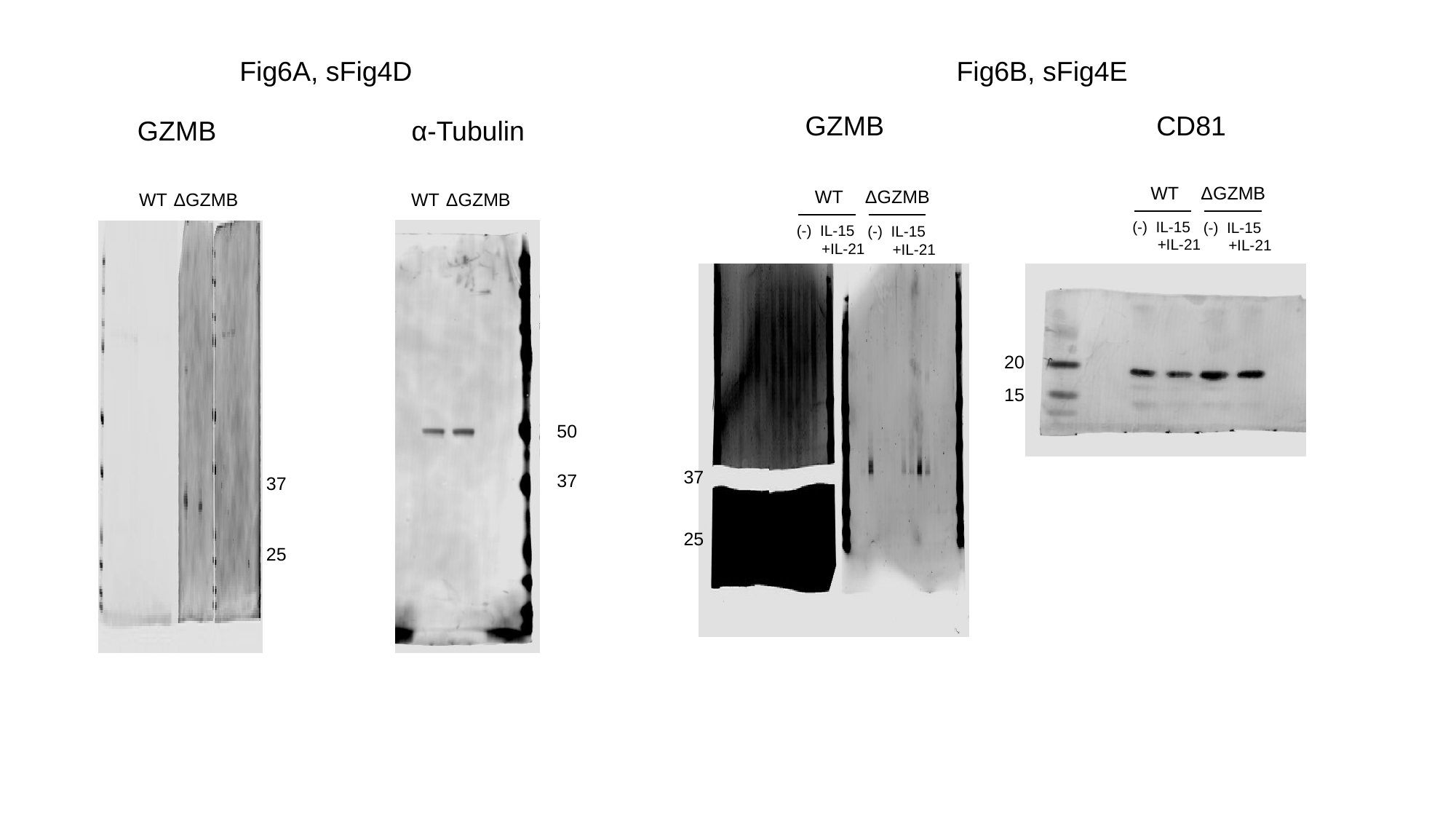

Fig6A, sFig4D
Fig6B, sFig4E
GZMB
CD81
GZMB
α-Tubulin
WT
ΔGZMB
WT
ΔGZMB
WT
ΔGZMB
WT
ΔGZMB
(-) IL-15
 +IL-21
(-) IL-15
 +IL-21
(-) IL-15
 +IL-21
(-) IL-15
 +IL-21
20
15
50
37
37
37
25
25
